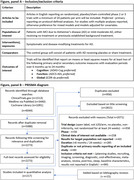# Systematic review and meta‐analysis of Alzheimer’s disease clinical trial results over more than a quarter century (1994‐2025) ‐ overall evidence and time savings

**DOI:** 10.1002/alz70859_100006

**Published:** 2025-12-25

**Authors:** Samuel P. Dickson, Benjamin A Haaland, Kent Hendrix, Patrick P O'Keefe, Newman Knowlton, Craig Mallinckrodt, Suzanne B. Hendrix

**Affiliations:** ^1^ Pentara Corporation, Salt Lake City, UT USA

## Abstract

**Background:**

Alzheimer’s disease (AD) trials led to recent successes with monoclonal antibodies targeting amyloid, opening up new directions for research into treatment for AD. Greater understanding of AD successes and failures will help researchers focus their efforts on the highest potential benefit, including combination treatments.

**Methods:**

We systematically reviewed placebo/sham‐controlled randomized clinical trials of symptomatic or disease‐modifying treatments for patients with mild cognitive impairment due to AD or mild‐moderate AD. Study results were included if at least two of the cognitive, function, and global domains were reported. Detailed inclusion criteria are provided in Figure, panel A. Time savings on a global statistical test (GST) combining cognitive, functional, and global domains assessed overall efficacy. The GST provides a stable measure of overall efficacy, while time savings provides an interpretable metric for magnitude of treatment benefits. Disease modifying and symptomatic effects across disease symptoms perform better on this GST than treatments impacting only one symptom. False positives are less likely to occur in three domains simultaneously, making the GST more reliable for detecting true treatment effects. Composite scores, ADCOMS, and iADRS were used to target true disease progression in lecanemab and donanemab phase 2 studies, and have similar advantages to GSTs. A similar approach may have averted much of the controversy surrounding the aducanumab approval.

**Results:**

Database searches yielded 6044 records. After exclusion of records not meeting inclusion criteria, duplicate reports, and non‐primary results of included trials, and inclusion of additional reports meeting inclusion criteria identified in bibliography reviews, there were 217 studies included in quantitative analyses ‐ including >130 AD treatments. Full PRISMA diagram is provided in Figure, panel B. Some studies previously determined failures show some indication of positive effects. Conversely, some studies previously thought promising are shown to be more clearly failures. Meta‐analyses combining similar treatments across programs illuminate overlooked mechanisms as well as more conclusive failures.

**Conclusions:**

Success and failure of Alzheimer’s treatments is partly obscured by including only one of three domains of clinical efficacy: cognition, function and global. True treatment efficacy and true lack of efficacy are much easier to detect with combined outcomes.